# What’s the uptake? Pragmatic RCTs may be used to estimate uptake, and thereby population impact of interventions, but better reporting of trial recruitment processes is needed

**DOI:** 10.1186/s12874-017-0443-0

**Published:** 2017-12-22

**Authors:** Katy J. L. Bell, Amanda McCullough, Chris Del Mar, Paul Glasziou

**Affiliations:** 1 School of Public Health, The University of Sydney, Camperdown, NSW 2006 Australia; 2 Centre for Research in Evidence Based Practice (CREBP), Bond University, Gold coast, QLD 4229 Australia

**Keywords:** Health policy, Public health, Primary health care, Pragmatic clinical trial, Methods, Drug resistance, microbial

## Abstract

**Background:**

Effectiveness of interventions in pragmatic trials may not translate directly into population impact, because of limited uptake by clinicians and/or the public. Uptake of an intervention is influenced by a number of factors.

**Methods:**

We propose a method for calculating population impact of clinical interventions that accounts for the intervention uptake. We suggest that population impact may be estimated by multiplying the two key components: (1) the effectiveness of the intervention in pragmatic trials (trial effect); and, (2) its uptake in clinical practice. We argue that participation rates in trials may be a valid proxy for uptake in clinical practice and, in combination with trial effectiveness estimates, be used to rank interventions by their likely population impact. We illustrate the method using the example of four interventions to decrease antibiotic prescription for acute respiratory infections in primary care: delayed prescribing, procalcitonin test, C-Reactive Protein, shared decision making.

**Results:**

In order to estimate uptake of interventions from trial data we need detailed reporting on the recruitment processes used for clinician participation in the trials. In the antibiotic prescribing example, between 75 and 91% of the population would still be prescribed or consume antibiotics because effective interventions were not taken up. Of the four interventions considered, we found that delayed prescribing would have the highest population impact and shared decision making the lowest.

**Conclusion:**

Estimates of uptake and population impact of an intervention may be possible from pragmatic RCTs, provided the recruitment processes for these trials are adequately reported (which currently few of them are). Further validation of this method using empirical data on intervention uptake in the real world would support use of this method to decide on public funding of interventions.

## Background

The effectiveness of an intervention in real clinical practice may be estimated in pragmatic trials conducted on patients who represent the full spectrum of the population to which the intervention might be applied, and where the comparator group receives usual care [1, 2]. Pragmatic trials are designed to determine the effects of an intervention under the usual conditions in which it will be applied, [3] and focus on the choice between options for care rather than biological understanding [4]. They may be contrasted to explanatory trials designed to determine the effects of an intervention under the ideal conditions, [5] in order to test causal research hypotheses, such as whether the intervention causes a particular biological effect [4]. Tools have been developed to help trialists with design decisions on how pragmatic or explanatory they wish their trial to be, [5, 6] and an extension of the CONSORT statement guides the reporting of pragmatic trials [7].

Estimates of intervention effectiveness may then be made using an intention to treat analysis of outcomes in the intervention group compared to those in the usual care group (‘trial effectiveness’) [7]. That is, patients are analysed in the group to which they were initially randomised, even if they drop out of the study or change groups [8]. But within trial effectiveness do not translate directly into population impact [9]. One important difference is uptake by clinicians and/or the public [10]. For example, for the long-term follow-up after colorectal cancer treatment, colonoscopy is the most sensitive method. But the less sensitive faecal occult blood testing has greater population impact because patients are willing to complete the four rounds of testing – that is, it has higher uptake [11]. Uptake of an intervention is influenced by a number of factors; for example for clinician uptake, these range from knowledge of the intervention and the skills to implement it, through to emotion regulation and beliefs about the intervention itself [12].

In this paper, we propose a method for calculating population impact of clinical interventions that accounts for the intervention uptake. We suggest that population impact may be estimated by multiplying the two key components: (1) the effectiveness of the intervention in pragmatic trials; and, (2) its uptake in clinical practice. That is,

population impact = trial effectiveness x rate of uptake.

Obtaining the trial effectiveness is straightforward from either primary pragmatic randomised controlled trials (RCTs) or meta-analyses of pragmatic RCTs. However, obtaining the uptake of an intervention by clinicians is more difficult. The best estimates come from studies measuring this directly (such as surveys of clinicians or patients), but these are rare [13]. An alternative indirect approach is to use data derived from the recruitment process of relevant pragmatic RCTs. Below, we illustrate how this can answer the following question relevant to a public health problem:

“*What intervention to minimise antibiotic prescribing for acute respiratory infections in primary care is likely to have the largest effect at a population-level?*”

## Example using RCTS to estimate population effectiveness

Interventions to minimise antibiotic prescriptions for acute respiratory infections in primary care.

### Method

Meta-analyses of pragmatic trials show that 4 interventions reduce antibiotic prescribing and/or consumption for acute respiratory infections in primary care:

- Delayed prescribing,
- Procalcitonin test
- C-Reactive Protein,
- Shared decision making

We calculated the population impact of each intervention using a 3-step process:

First, we extracted the meta-analytic estimates of the *trial effectiveness* of the interventions (two extractors). Where these were not presented in the original meta-analysis, we undertook new analyses in RevMan 5.3 [14] to obtain relative risks (with random effects models) and confidence intervals.

Second, we calculated the rate of *uptake*. From each systematic review we identified pragmatic RCTs that reported the number of clinicians invited to particpate, and those who did participate. We calculated the uptake by dividing the number of GPs who participated by those invited. Where this was reported for both GP practices, and GPs within participating practices, we calculated uptake by multiplying the two proportions. We excluded RCTs from this analysis that did not have a population based recruitment (e.g. only invited academic GPs attached to a University department). Where there was more than one RCT for estimating the uptake, we estimated the mean uptake rate and confidence intervals using a logistic regression model with random intercepts, using PROC NLMIXED in SAS software, version 9.4 [15]. Where there was only one RCT, we calculated confidence intervals for a single proportion using the binomial distribution.

Finally, we calculated *population impact* of the intervention by multiplying the trial effectiveness by the uptake.

### Results

Table 1 summarises the 4 included systematic reviews that provided estimates of trial effectiveness and the 6 pragmatic RCTS (1 for delayed prescribing [16], 1 for procalcitonin [17], 1 for C-reactive protein [CRP] [18], and 3 for shared decision making [19–21]) that provided estimates of uptake. All reviews were on patients with acute respiratory infections, and the RCTs were conducted across several countries including Denmark, Germany and Canada. The RCTs were all primarily of pragmatic type, with high applicability to the actual clinical settings the intervention was intended to be used, and a very good match between the trial usual care arm and the intended primary care population [6].

**Table 1:** Characteristics of included systematic reviews for estimating trial effectiveness, and of RCTs for estimating uptake, of interventions to decrease antibiotic prescription for acute respiratory infections in primary care

	Systematic Reviews used for estimating trial effectiveness		Pragmatic RCTs used for estimating uptake	
Intervention	Clinical population	Outcome	Clinical population	Recruitment of clinicians
Delayed prescribing [25]	People patients of all ages with upper respiratory tract infection	Antibiotic use (i.e. prescriptions filled)	Adults with acute cough, for whom the physician intended to prescribe antibiotics [16]	22/61 practices approached actually participated. 48/92 GPs recruited patients to the trial (range = 1–25 per GP).
Procalcitonin [26]	Adults with an acute respiratory infection (including lower respiratory infection such as pneumonia).	Antibiotics prescribed	Adults with an acute respiratory infection, for whom the physician intended to prescribe antibiotics [17]	53/345 eligible primary care physicians in 2 cantons in northwest Switzerland actually participated
CRP [27]	People of all ages with acute respiratory infection (including lower respiratory infection such as pneumonia)	Antibiotics prescribed	People of all ages with respiratory Infections (median age = 37 years [18])	35/125 GPs in single-handed practices in the County of Funen actually participated
Shared decision making [28]	People of all ages with acute respiratory infection (including lower respiratory infection such as pneumonia)	Antibiotics prescribed, dispensed or decision to use	Adults with acute respiratory infection. [19]	45/345 eligible GPs in two cantons, Basel-Stadt and Aargau actually participated.
			People of all ages with acute cough (mean age = 42 years [20]	101/2036 GPs from 9 regions in North-Rhine and Westphalia-Lippe actually participated
			People of all ages with respiratory Infections (71% adults) [21]	4/24 Family Medicine Groups (FMGs) in Quebec actually participated. Within the participating FMGs, 33/42 FPs participated

*CRP* C-Reactive Protein, *GP* General Practitioner, *FMG* Family Medicine Group, *FP* Family Practitioner

Delayed prescribing had the highest *trial effectiveness* (relative risk reduction [RRR] of 64% in antibiotic consumption) and CRP the lowest (RRR 22% in antibiotics prescribed) (Table 2). New meta-analytic estimates were calculated for procalcitonin (RRR 61%) and delayed prescribing (RRR 64%). There was substantial heterogeneity between studies for both of these interventions (RRR range 42–74% for procalcitonin and 42–79% for delayed prescribing).

**Table 2:** Estimated trial effectiveness, uptake and population impact of four interventions to decrease antibiotic prescription or consumption for acute respiratory infections in primary care

	Trial effectiveness				Uptake of intervention			Population impact	
Intervention	Total event rate in intervention group	Total event rate in control group	Summary Relative Risk (95% CI)	Relative Risk Reduction (RRR;1-RR)	Total number of participating GPs	Total number of potential participating GPs	Population Uptake (95% CI)	Relative risk reduction (RRR × Uptake)	Ranking
Delayed prescribing	255/817	790/847	0.36 (0.27–0.48)	64%	48	255^d^	19% (14–24%)	12.0%	1
Procalcitonin	117/507	320/501	0.39 (0.17–0.86)	61%	53	345	15% (12–19%)	9.4%	2
CRP	631/1685	785/1599	0.78 (0.66–0.92)	22%	35	125	28% (20% – 36%)	6.2%	3
Shared decision making	5236^a^	4936^a^	0.61 (0.55–0.68)	39%	281^b^	3389^b^	9.5% (0–20%)^c^	3.7%	4

^a^Number of events not reported

^b^Total across 3 RCTs, combined in a logistic regression model with random intercepts

^c^Lower CI set as ≥0

^d^22/61 practices participated; out of participating practices 48/92 GPs participated; therefore number of potential participating GPs is 92 × (61/22) = 255

In contrast, CRP had the highest *uptake* rate (28%) and shared decision making the lowest (10%) (Table 2), meaning that between 72 and 90% of clinicians invited to participate did not wish to use these interventions. (We pooled data from 3 RCTS to estimate population uptake for shared decision making, and used data from 1 RCT to estimate uptake for the other 3 interventions).

**Fig. 1:**
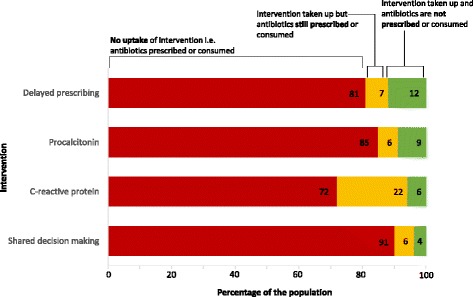
Estimated population impact for four interventions that aim to reduce antibiotic use for acute respiratory infections. Estimates are for a patient population who would be prescribed antibiotics in absence of intervention

Delayed prescribing had the highest *population impact* at 12% and shared decision making the lowest (4%) (Table 2, Fig. 1). For delayed prescribing, this means that if implemented, in 12% of clinical encounters where a person with an acute respiratory infection would otherwise have been prescribed antibiotics, the doctor would *use (uptake) the intervention and the patient would not consume antibiotics* (Table 2, Fig. 1), in 7% the doctor would *use the intervention but the patient would still consume antibiotics*, and in 81% the doctor would *not use the intervention and the patient would consume antibiotics.*

The most notable finding is that the greatest constraint to population impact is the poor uptake of interventions by clinicians rather than the effectiveness of the interventions themselves. Between 72 and 91% of the population would still be prescribed or consume antibiotics because effective interventions have not been taken up, Fig. 1. Of the four potential interventions shown to decrease antibiotic prescribing in RCTs, delayed prescribing is the intervention with the highest population impact, and could prove the best choice for implementation.

## Challenges to this method

### Better reporting of trial recruitment is needed

The poor reporting of both the uptake by clinicians, and of the methods of recruitment, limit our ability to estimate overall impact and hence inform health policy. In the antibiotic prescribing example, only some trials reported the number of general practices randomised out of those approached, and only a few reported on the number of clinicians who then participated out of the total eligible within each practice. No trials reported on the number of patients randomised out of the total number of eligible patients seen by each of the participating GPs.

Better reporting of both recruitment rates and recruitment methods are crucial to new RCTs. The extended CONSORT checklist [6] and flow diagram requires reporting of eligibility criteria to show the degree to which they include typical participants and/or typical providers, institutions, communities and settings of care, and the number of participants or units approached to take part in the trial, the number which were eligible, and reasons for non-participation [22]. We recommend that criteria be added to this to include reporting on the methods of recruitment, including the dialogue and incentives, and the resulting recruitment rates. In this way, we can utilise data which are routinely collected in pragmatic trials but not currently publicly available, helping to reduce waste in research [23]. Just as estimates of trial effectiveness may be context-dependent, so too may be the trial estimates of uptake, and results may not be easily translated across different settings. Ideally, estimates of uptake should be based on meta-analysis of multiple trials of the intervention, with exploration of heterogeneity between trials to identify potential facilitators and barriers of intervention uptake. This would allow estimates of population impact under best case and worse case scenarios as well as helping inform implementation strategies. The actual population impact may be lower than in these scenarios, as even when the clinician and patient decide to uptake the intervention, they are generally less adherent to this in real life than in the trial setting (and so the trial effectiveness overestimates the real life effectiveness).

### Does uptake in RCTs predict uptake in clinical practice?

The uptake reported in pragmatic RCTs may be either under- or over-estimates: the additional perceived burden of the research element in RCTs may lead us to underestimate uptake; however, the extra support provided in the research setting may result in overestimates of uptake in the real world. These biases may favour one intervention over another. A further issue is that the willingness of clinicians to participate in pragmatic trials where the effectiveness of an intervention is unknown, may be different to the uptake in clinical practice when effectiveness has been established. For instance, interventions that have been shown to be effective may be promoted or even mandated in ways which would affect participation in practice. However, in the early pre-implementation stage where we are suggesting the method may be used to inform funding decisions (i.e. after the completion of the trials, but before a decision to publically fund and/or promote the intervention has been made), these considerations may not be as important.

Validation of our method may be possible for interventions that have previously received public funding. For example, a large prospective cohort study found that 14% of UK based general practitioners used delayed prescribing of antibiotics for adults presenting with a sore throat [13] – this is not dissimilar to our estimated uptake rate of 19% (95% CI 14–24%). We could not find population based studies on uptake of the other three interventions in clinical populations similar to those in trials. One nationwide cross-sectional study found that 70% of Norwegian GPs used CRP for children aged 5 years or under who presented with a respiratory infection [24], but use of CRP for adult patients is not known. We estimated that CRP had an uptake rate of 28% (95% CI 20–36%) for use in adults, and although this was the highest uptake out of the 4 interventions we studied, it may still be an underestimate. Prospective evaluation as part of implementation trials could also help us work out if our uptake estimates are reflective of “real-life” uptake rates. If such comparisons support the use of our method for ranking interventions on their likely uptake in clinical practice, then this method might also be used to decide on public funding of interventions at the outset.

## Conclusions

For estimating the population impact of interventions, knowing the uptake rate may be as important as the trial effectiveness, but is not usually considered when deciding on public funding. Estimates of uptake may be possible from pragmatic RCTs of the intervention, provided the recruitment processes for these trials are adequately reported (which currently few of them are).

## Abbreviations

CONSORT: Consolidated Standards of Reporting Trials
CRP: C-reactive protein
GP: General Practitioner, equivalent to Primary Care Physician
RCT: Randomised Controlled Trial
RRR: Relative risk reduction
UK: United Kingdom
